# A research update on the state of play for return to sport after anterior cruciate ligament reconstruction

**DOI:** 10.1186/s10195-018-0516-9

**Published:** 2019-01-28

**Authors:** Kate E. Webster, Julian A. Feller

**Affiliations:** 1 School of Allied Health, College of Science, Health and Engineering, La Trobe University, Melbourne, VIC 3086 Australia; 2 OrthoSport Victoria, Epworth HealthCare, Melbourne, Australia

**Keywords:** Return to sport, Knee injury, Athlete, ACL, Reconstruction surgery

## Abstract

Most athletes who undergo anterior cruciate ligament (ACL) reconstruction surgery plan to return to some level of sporting activity. However, rates of return to pre-injury sport are often less than might be expected and many factors influence whether individuals return to sport after this surgery. They include surgical and rehabilitation factors as well as social, psychological and demographic characteristics. The fate of the younger athlete who sustains an ACL injury is a topic that has received recent attention due to accumulating evidence that younger athletes are at considerable risk for not only one, but multiple ACL injuries. Little is known about how to determine when it is safe to return to sport following ACL reconstruction or how to predict whether an athlete will be able to successfully return. The notion that a set of return to sport criteria can be applied to reduce the risk of further injury has become popular with many different criteria proposed. Another risk of returning to sport following ACL reconstruction is that of sustaining injury to the menisci or articular surfaces, which may in turn increase the risk of developing osteoarthritis. Although there is some evidence that ACL reconstruction reduces the risk of osteoarthritis there is stronger evidence that it does little to protect the knee from long term degeneration. Therefore, it should be recognized that return to sport following ACL reconstruction is associated with a risk of further injury and potential development of osteoarthritis.

**Level of evidence**: V

## Introduction

The goal for most athletes who sustain an anterior cruciate ligament (ACL) injury and elect to undergo reconstruction surgery is to be able to return to their preinjury sport. However, over the past decade it has become apparent that the rates of return are less than ideal and certainly less than what might be expected from standard activity and impairment-based measures [1, 2]. Return to sport after ACL reconstruction has therefore became a focus of the research literature. The purpose of this article is to summarise and explore the current knowledge regarding return to sport after ACL reconstruction surgery.

## Return to sport terminology

One fundamental question is what constitutes a return to sport? Whilst this may initially seem straightforward to answer, it is complicated as terminology has varied. For example, is a return to any kind of activity (such as a weekend run) considered to be a return to sport in the same way as return to strenuous activity that involves cutting and pivoting movements (such as basketball or soccer)? Clearly the latter is more demanding on knee function and increases the athlete’s risk for future knee injury. This means that precise terminology is important.

Whilst there is yet to be consensus on the preferred terminology, two papers have attempted to provide working definitions [3, 4]. Lynch et al. [3] initially defined return to sport as “one or two seasons in the sport of injury at the same level as prior to injury”. More recently a consensus statement described a return to sport continuum [4]. The continuum has three elements, the first being a return to participation, the second a return to sport and the third a return to performance. *Return to participation* may be a return to training or a level of sport that is lower that what the athlete desires. A *return to sport* is designated as the athlete having returned to their pre-injury sport of choice but not performing at the desired level, whereas a *return to performance* signifies that the athlete is now performing at or above the preinjury level in their chosen sport. Empirical application of this theoretical continuum has however not yet been validated for return to sport after ACL reconstruction.

## Return to sport rates

An initial systematic review with meta-analysis determined the rate of return to any kind of sports participation as well as the rates of return to pre-injury and competitive sports following ACL reconstruction surgery [5]. Results from 48 studies that reported on outcomes in 5770 patients showed that overall, 82% of patients returned to some kind of sport, but only 63% were participating in their pre-injury sport at follow-up. When competitive sport was considered, only 44% were participating at follow-up. These participation rates contrasted with the finding that around 90% of patients were rated normal or nearly normal on impairment-based outcomes such as strength and knee laxity. This review was updated in 2014 to include a total of 69 studies reporting on 7556 patients [6]. In the update, 81% returned to some kind of sport, 65% returned to their pre-injury sport and 55% returned to competitive sport. The overall message was that return to sport rates are less than might be expected by an athlete undergoing ACL reconstruction.

A review of return to sport rates that focussed on younger patients aged 6 to 19 years was recently conducted [7] and included 20 studies on 1156 patients. Ninety-two percent returned to some kind of sport, 79% to pre-injury sport and 81% to competitive sport. Return to sport rates are therefore notably higher for younger athletes and this has implications for reinjury which will be later discussed.

Most of the patient cohorts in the above reviews had undergone primary ACL reconstruction. Whilst there is less information regarding return to sport rates after revision reconstruction, a 2015 systematic review by Grassi et al. [8] which included 23 studies reporting on 1090 patients and showed similar results to the primary ACL reconstruction reviews. Eighty-five percent returned to some kind of sport, 53% to the pre-injury level and 51% to competitive sports. The review is however limited by the availability of only 4 studies in competitive athletes and only two studies with larger (> 100) patient numbers. A more recent study has also shown higher return to competitive sport rates (68%) after revision reconstruction in adolescent athletes [9].

There is a paucity of information regarding return to sport for athletes who have had ACL reconstructions to both knees. The limited studies show that a high proportion (70–80%) return to sport after the first reconstruction procedure but that return rates drop dramatically to less than half after a second (contralateral) reconstruction procedure [10, 11]. The timing between surgeries does not appear to affect return rates, with patients who have two procedures within 3 years just as likely to cease sport participation as those that have a longer time interval between surgeries [11].

Perhaps not surprisingly, elite level athletes have been shown to have the highest rates of return to pre-injury levels of sport. A systematic review of 24 studies reporting on 1272 elite level athletes showed 83% returned to their pre-injury sport [12]. Most of studies in the review reported that their elite athlete cohort had returned within 12 months of surgery, with only two studies in American football players reporting longer than 12 months on average to return [13, 14]. Return rates after a second ACL injury are also relatively high (71%) in elite level athletes [15].

### Factors that influence return rates

There are many factors which influence whether an individual will return to sport after ACL reconstruction surgery. They include surgical and rehabilitation factors as well as social, psychological and demographic characteristics [16]. Factors for which there is supporting empirical data include being male and of younger age. On average males tend to have higher return rates by approximately 10% and the return rates for younger patients can be in the order of 30% higher than older aged patients [5, 6, 17]. Patients who experience a shorter time interval between injury and surgery have higher return rates and, as already noted above, playing elite level sport favours a return [12]. Results in terms of graft type have been mixed with hamstring tendon autografts favouring a return to competitive sport at various levels and patellar tendon autografts favouring a return to pre-injury sport, although this discrepancy may reflect definitions and terminology used in different studies [6]. Having a positive psychological response has been shown to be strongly associated with a return to pre-injury sport [18–22]. Higher levels of motivation during rehabilitation have also recently been shown to be associated with higher rates of return to preinjury sport following ACL reconstruction [23].

## Reasons why athletes don’t return or give up sport

From all the data summarised thus far it is clear that return to sport rates following ACL reconstruction are less than might be anticipated. The role of patient aspirations and expectations should therefore be considered as it is possible that some patients simply do not expect to return to their pre-injury sport. However, this does not seem to be the case. An initial study by Feucht et al. [24] showed that 91% of athletes expected to be able to return to the same level of sport. A more recent study in a large cohort of 675 patients found that 84% expected to be able to return to their previous level of sport [25]. This cohort had all participated in high level competitive or frequent sports prior to injury. It was further shown that expectations were higher for patients about to undergo their first ACL reconstruction, where 88% expected to return, than for those about to undergo revision surgery or a second primary ACL reconstruction (63% and 80% respectively).

Many factors are likely to influence a patient’s expectations and importantly expectations may change after surgery. At 12-month post-surgery it was shown that 15% of the patient cohort had already decided to give up sport, with females and patients who had undergone a previous ACL reconstruction most likely to change their expectations and cease sport participation [25].

Perhaps the most commonly cited reason by athletes for not returning to sport after ACL reconstruction surgery is fear of reinjury. Kvist et al. [26] initially identified fear of re-injury as a significant factor in patients who did not return to their previous level of activity after ACL reconstruction. This finding has been supported and quantified by numerous subsequent studies with some showing that up to 50% [27] of athletes who do not return to sport cite fear of re-injury as the reason. Restriction of sporting activity due to fear of re-injury has also been reported [28]. Exactly what constitutes fear of re-injury is unclear. It may be fear of the pain of injury itself, fear of the implications for time off work and the related loss of income, fear of not being able to return to the previous level of function, or any combination of these.

Not trusting the knee and having poor self-reported knee function have also been cited as reasons for not returning to sport after ACL reconstruction [29]. For athletes who return to sport but subsequently cease participation, work or study commitments are cited as the most common cause for stopping [30].

## Sport performance following ACL reconstruction

It is important to consider performance as well as simply returning to the field or court. Consideration should also be given to whether assessment of performance is based on the athlete’s perception or on documented metrics. Despite return to performance being highlighted as the final stage in the return to play continuum, there is relatively little empirical data to determine whether athletes can return to their pre-injury level of performance following ACL reconstruction surgery. The most reliable and valid way to measure performance is also highly debatable. A recent review which looked at return to sport specific performance following ACL reconstruction surgery showed that whilst most high-performance and professional athletes returned to their preinjury level of sport there were measurable decreases in performance statistics [31]. These decreases tended to be highly sport-specific. The review noted the paucity of available literature on this topic and cautioned that the existing literature is highly biased and must therefore be read with caution.

For non-elite younger athletes, approximately two-thirds self-report being able to play at their pre-injury level of performance at 2 years after their reconstruction surgery [30]. Interestingly, patients who rate their ability to perform as the same as before their injury are also more likely to continue to participate in their pre-injury sport for more years following surgery [30]. It is currently unclear exactly what factors contribute to a patients self-reported rating of performance. However, most patients who return to their preinjury level of sport report similar levels of performance compared to before their injury, unlike those who return to a lower level of sport [11].

## When should athletes return?

Perhaps the most difficult question to answer is when is it safe for the athlete return to sport? There are two issues we are concerned about in relation to this question; (1) graft rupture/failure, and (2) damage to the rest of the knee, both in the short and longer term. For the surgery to be considered truly successful, it should enable the patient to return to sport without further injury or damage to the knee.

From animal studies it has been established that there are distinct phases of graft maturation with early graft necrosis and subsequent hypercellularity and revascularization being the potential risk periods for re-injury [32]. In humans there is of course much less information. It is suggested that there are the same phases, but that they occur over a much slower time frame than in animals [33]. Whilst there are differences between the available studies, the period of remodelling which is where the graft is most at risk, seems to correspond to roughly the 4- to 12-month time point which also corresponds to the time when many athletes are returning to pivoting sports [33]. Particularly concerning is data from hamstring tendon autografts which suggest that remodelling may take up to 12 to 24 months, which is when the peak of second injuries seems to occur [34].

Returning to sport puts the individual at risk of both ACL graft rupture and rupture of the contralateral ACL. Overall, it has been shown that the risk for graft rupture is highest during the first two postoperative years whereas the risk for contralateral ACL injury appears to occur relatively later and increases in relative terms over time [35–37]. Numerous risk factors for re-injury have been investigated but without many consistent findings. Most studies have reported on sex and age. Sex as a risk factor for graft rupture has either shown no influence or males, particularly younger males, have been shown to be at greater risk [37–39]. In contrast, if there is a sex effect for subsequent contralateral ACL injury, females appear to be at greater risk [40, 41].

In recent years there has been a growth of evidence from both large cohort studies and registry databases to confirm that younger athletes are at significant risk for second ACL injury [42]. Specifically, cohort studies have shown that between 20 and 30% of younger athletes sustain a second ACL injury [36, 37, 43, 44]. It has also been reported that 27% of patients aged less than 25 years at the time of ACL revision surgery go on to have a third ACL injury [45]. Such statistics are indeed concerning, and it is important to better understand the reasons why younger patients are at such high risk. It is unlikely that age itself is the risk factor, but rather a proxy for multiple factors. The most salient of these factors is that younger patients are more likely to return to sport [7] and, when they do, the sports they play are high risk sports [7]. As such, the rehabilitation of and timing for return to sport needs to be carefully considered.

### Rehabilitation programs and return to sport criteria

There has been a marked interest and rapid growth in studies that document return to sport criteria. There are two broad approaches two addressing readiness to return to sport. One is to target deficits known to be associated with second ACL injury in phased rehabilitation programs and set criteria for progression from one phase to the next, including return to sport [46]. This is contrast to more traditional time-based protocols, which are based on our concept of what is happening in terms of graft maturation. The second approach, which can be applied to both types of rehabilitation, is to use a set of criteria or ‘test-battery’ to ‘clear’ the athlete for return to sport. This is typically used at the final phase of rehabilitation and athletes who ‘pass’ are cleared to return.

Several consensus statements have recently been published with the aim of determining return to sport criteria. van Melick et al. [47] attempted to reach a consensus regarding which criteria should be used to determine the moment of return to play. It was recommended that an extensive test battery for both quantity and quality of movement should be performed. As a minimum, the test battery should include a series of strength tests, hop tests and measurement of quality of movement. A limb symmetry index of greater than 90% was suggested as a pass criterion, but it was also suggested that this could be increased to 100% for patients planning a return to pivoting or contact sports. An additional consensus statement concluded that, for any injury, the return to sport decision should always use information gained from a battery of tests and should assess direction change and reactive agility, as well as psychological readiness [4].

It is interesting that the more recent studies have attempted to cover a broad range of risk factors, often including 15–20 return to sport tests [48, 49]. This is likely due to the lack of clear evidence as to what are the most important risk factors for reinjury or, indeed, whether the tests are designed to determine whether the patient is capable of returning to play or whether they are designed to determine whether it is safe. Perhaps the focus should be on fewer but important risk factors for reinjury, and it has been suggested that five factors should be sufficient as any one factor would ideally account for at least 20% of the predictive variance [50]. The proportion of patients that are reported to pass test batteries is typically low, and whilst there is considerable variation between studies, pass rates of less than 30% are most commonly reported [51–55]. From a practical standpoint, such low pass rates should raise the question of how such tests can be utilized if a majority of patients fail.

Five prospective studies have been conducted to investigate the association between return to sport testing and subsequent injury [56–60]. Two of these have shown a significant effect of passing criteria and subsequent ACL injury, but with conflicting results. Krystis et al. [58] recorded graft ruptures in elite male athletes and reported that those who did not meet all return criteria had a four times greater risk of graft rupture than those who did. In comparison, Sousa et al. [59] did not find a reduced risk for graft rupture in their group who passed criteria, but they did find a significantly increased risk for contralateral injuries. Therefore, passing a return to sport test battery has on the one hand been shown to significantly reduce the risk of subsequent graft rupture, but on the other hand has also been shown to increase the risk of a contralateral ACL injury. As such, return to sport test batteries currently have limited validity in the reduction of overall second ACL injury risk.

### Risk of osteoarthritis

It is important to consider the health to the rest of the knee if an athlete returns to sport after ACL reconstruction. ACL injuries are rarely truly isolated injuries, with frequent bone bruising, meniscal damage and chondral lesions. An extensive bone bruise may take months to resolve, which raises the question of how quickly rehabilitation should be progressed. The concept of homeostasis of the joint is also relevant within this context and it seems reasonable that one should stay within the boundaries of homeostasis to reduce the risk of further damage [61].

A seminal review by Lohmander et al. [62] reported that approximately 50% of those who sustained an ACL injury developed osteoarthritis at an average of 10 years post injury. A subsequent review showed that the rate of osteoarthritis following ACL injury was substantially less if there was no associated meniscal damage [63]. Whether ACL reconstruction can prevent the development of osteoarthritis is highly debatable and on balance the recommendation for surgery for prevention of osteoarthritis cannot be supported. A systematic review showed that 44% of 2500 patients who had reconstruction surgery developed osteoarthritis compared to 37% of 337 patients with ACL deficiency [64]. Interestingly, there was little difference in the rate of osteoarthritis between the groups during the first decade after injury or surgery with the greatest difference in rates occurring during the second decade. It was suggested that reconstruction surgery may have permitted a return to activity after ACL reconstruction and this may have increased the subsequent risk for the development of osteoarthritis.

An interesting finding in terms of return to sport and the development of osteoarthritis was recently published and showed that patients who had returned to pivoting sports had a 72% reduction in the odds for developing symptomatic osteoarthritis and a 60% reduction in the odds for radiographic osteoarthritis compared to patients who had not returned to pivoting sports at 15 years after ACL reconstruction surgery [65]. The reasons for this difference are however unclear and it is relevant to note that 59% of the group who failed to return to pivoting sports also reported poor knee function, which may have acted as a confounding variable. The most recent review of the rates of osteoarthritis after ACL reconstruction surgery reports rates of 11% at 5 years, 21% at 10 years, and 52% at 20 years [66]. The rates were higher with an increased time interval from injury to surgery, but the role of activity was unfortunately not evaluated [66].

## Conclusions

In conclusion, the rates of return to sport following ACL reconstruction are often less than might be expected and many factors influence a return to play. Younger athletes have high return to sport rates and higher rates of further ACL injury. There are currently no validated guidelines for knowing when it is safe to return. There is some utility in return to sport criteria, and they can be used to provide the patient with feedback with regard to their rehabilitation progress. However their overall application when it comes to clearing an athlete to return to sport in clinical practice is unclear due to a high level of uncertainly as to their validity regarding the risk for subsequent injury. Ultimately it must be recognised that any return to sport is associated with a risk of further injury and potentially the development of osteoarthritis.

## Abbreviation

ACL: anterior cruciate ligament
